# A Novel Method for Predicting Ideal Postoperative Upper Instrumented Vertebra Tilt to Prevent Lateral Shoulder Imbalance after Scoliosis Correction Surgery

**DOI:** 10.3390/jpm13030393

**Published:** 2023-02-23

**Authors:** Wen Zhang, Mengmeng Xu, Weimin Zhang, Tao Li, Yudong Lai, Fei Chen, Mingtong Sun, Haoyu Wang, Jianmin Sun, Xingang Cui, Zhensong Jiang

**Affiliations:** 1 Department of Spine Surgery, Shandong Provincial Hospital Affiliated to Shandong First Medical University, Jinan 250021, China; 2 Department of Pediatrics, Qilu Hospital of Shandong University, Jinan 250012, China

**Keywords:** lateral shoulder balance, upper instrumented vertebra tilt, clavicle angle, scoliosis, correction surgery

## Abstract

Lateral shoulder imbalance (LSI) is reflected radiologically by the clavicle angle (CA). How to achieve postoperative lateral shoulder balance (LSB) after scoliosis correction surgery remains unclear. In the current study, by using the preoperative upper instrumented vertebra (UIV) tilt, the CA, the flexibility between T1 and the UIV, and the ideal postoperative UIV tilt was predicted based on the following formula: ideal postoperative UIV tilt = preoperative UIV tilt—the flexibility between T1 and UIV—preoperative CA. The reliability of the formula was verified through a retrospective analysis, and 76 scoliosis patients were enrolled. The feasibility of this method was verified through a prospective analysis, and 13 scoliosis patients were enrolled. In the retrospective study, there was a significant correlation between the difference in the actual and ideal postoperative UIV tilt values and the postoperative CA, with correlation coefficients in the whole, LSI, and LSB groups of 0.981, 0.982, and 0.953, respectively (*p* < 0.001). In the prospective study, all patients achieved satisfactory LSB. Using the formula preoperatively to predict an ideal postoperative UIV tilt and controlling the intraoperative UIV tilt with the improved crossbar technique may be an effective digital method for achieving postoperative LSB and has important clinical significance.

## 1. Introduction

Shoulder balance, as measured by the Walter Reed Visual Assessment Scale (WRVAS), is one of the most commonly rated specific appearance concerns described by scoliosis patients and has shown validity as a means for assessment of scoliotic deformity. Postoperative shoulder imbalance (PSI) is an important complication of scoliosis correction surgery [1]. Although many spine surgeons have conducted many studies on PSI, the incidence is still high, ranging from 25% to 57% [1,2]. Previous studies have reported that although patients with PSI did not require revision surgery, PSI immediately after surgery should be avoided because it may not only affect the patients’ appearance but may aggravate the postoperative adding-on (one of the frequent postoperative complications occurring at the segment distal to the lower instrumented vertebra, LIV, which often results in unsatisfactory radiographical and clinical outcomes including correction loss, wedging, and degeneration of the adjacent disc, and coronal decompensation) or trunk shift phenomenon (the distance between the C-7 plumb line and the central sacral vertical line > 20 mm) during the follow-up period [3]. Therefore, it is necessary to optimize shoulder balance during correction maneuvers intraoperatively to prevent these problems.

There are two distinct types of PSI: medial shoulder imbalance (MSI) and lateral shoulder imbalance (LSI) [4,5]. MSI is reflected radiologically by T1 tilt (measured by the angle subtended by a line drawn along the cephalic endplate of T1 and a horizontal reference line), the first rib angle (FRA, the tilt of the tangential line that connects both the superior borders of the first ribs), and the degree of proximal thoracic (PT) curvature [6,7], and LSI is reflected radiologically by the clavicle angle (CA, the intersection of the line connecting the highest two points of each clavicle and horizontal line) [8]. Lateral shoulder balance is also called clinical shoulder balance, and how to achieve laterally balanced shoulders postoperatively remains unclear [8,9]. Current studies in the literature have reported that radiographic parameters, such as T1 tilt, which better correlates with MSI but correlates poorly with LSI, cannot be used as an intraoperative proxy for lateral shoulder balance (LSB) [10,11]. Similarly, our study found that although there were weak or no significant correlations between T1 tilt and the CA both pre- and postoperatively, the change in T1 tilt after correction surgery was significantly related to the change in the CA, which suggests a close connection between T1 and the clavicle.

However, in clinical work, we do not routinely fuse T1 but instead fuse T2 or below [9,12]. The importance of selecting the proper upper instrumented vertebra (UIV) to prevent LSI has been debated in many previous studies [13,14]. However, none of these UIV selection systems can accurately predict the occurrence of LSI [15,16]. Since there is a close correlation between the change in T1 tilt and CA after correction surgery, maintaining a proper postoperative T1 tilt may be helpful to achieve LSB. To achieve this, the preoperative CA and any factors that may affect the postoperative T1 tilt should be considered. Previous studies have shown that when UIV is at T2, the correlation between UIV tilt and T1 tilt is higher than when UIV is at T3 or T4, which may be related to the flexibility between UIV and T1 [15]. Therefore, the postoperative T1 tilt may be affected by the preoperative UIV tilt and the flexibility between T1 and the UIV. The flexibility between T1 and the UIV is measured as follows: if the right side of the UIV is high before surgery and the left side needs to be raised, we need to take into account the maximum compensatory ability between T1 and UIV to bend to the left (the difference between the upper endplate angle of T1 and UIV in the AP position and left bending of the whole spine); if the UIV is high on the left side before surgery and the right side needs to be raised, we need to refer to the maximum compensatory ability between T1 and UIV to bend to the right (the difference between the upper endplate angle of T1 and UIV in the AP position and right bending of the whole spine). Taking the above factors into consideration, we predicted the ideal postoperative UIV tilt preoperatively using the following formula: ideal postoperative UIV tilt = preoperative UIV tilt—the flexibility between T1 and UIV—preoperative CA. Therefore, the purpose of this study was to verify the reliability of the formula and its effectiveness in clinical practice, which may provide a practical, digitally controllable method for achieving postoperative LSB.

## 2. Materials and Methods

### 2.1. Patient Population

In this retrospective study, after receiving approval from our institutional ethics committee review board (reference number: 2021–194), 76 scoliosis patients who underwent spinal fusion and instrumentation to correct their spinal deformity were recruited from our institution between October 2016 and April 2020. X-ray analysis revealed that PT curves were 33.9 ± 6.4° preoperatively and 17.3 ± 7.2° immediately postoperatively; MT curves were 56.4 ± 8.9° preoperatively and 23.5 ± 8.3° immediately postoperatively. In the prospective study, 13 scoliosis patients were enrolled at our institution between May 2020 and September 2021. PT curves of this group were 34.6 ± 7.5° preoperatively and 16.3 ± 8.5° immediately postoperatively; MT curves were 58.4 ± 9.9° preoperatively and 22.3 ± 7.8° immediately postoperatively.

### 2.2. Surgical Technique

All correction surgeries were performed at our hospital by a senior spine surgeon. The patient was placed in the prone position on the operating table. Paired alternate-level pedicle screws were placed with a freehand technique. The reduction maneuver consisted of concavity distraction, convexity compression, rod rotation, and segmented de-rotation techniques. In addition, if any residual curvature existed, in situ bending of the rod was performed to obtain maximal correction.

### 2.3. Radiographic Measurements

Standing and side-bending anteroposterior (AP) and lateral radiographs of the entire spine were obtained preoperatively, and standing AP and lateral radiographs were obtained immediately after surgery. The preoperative UIV tilt, preoperative CA, flexibility between T1 and the UIV, ideal postoperative UIV tilt, postoperative UIV tilt, and postoperative CA were measured [11,15] or calculated by the following formula:

ideal postoperative UIV = preoperative UIV tilt − the flexibility between T1 and UIV* − preoperative CA

* If the right side of the UIV is high before surgery and the left side needs to be raised, we need to take into account the maximum compensatory ability between T1 and UIV to bend to the left (the difference between the upper endplate angle of T1 and UIV in the AP position and left bending of the whole spine); if the UIV is high on the left side before surgery and the right side needs to be raised, we need to refer to the maximum compensatory ability between T1 and UIV to bend to the right (the difference between the upper endplate angle of T1 and UIV in the AP position and right bending of the whole spine).

For these measurements, a positive value indicates that the left side is higher, and a negative value indicates that the right side is higher [8]. All radiological parameters were measured by two attending spine surgeons who were not involved in the surgery and were averaged to obtain the final value.

Postoperative LSB was defined as |CA| < 3 degrees [17]. Our 76 patients were divided into three groups based on postoperative LSB: (1) the whole group, including the LSI and LSB groups (*n* = 76); (2) the LSI group (*n* = 26); and (3) the LSB group (*n* = 50).

### 2.4. Verification of the Feasibility of This Method in Clinical Practice

We used the crossbar method reported in the literature [18] and improved this method in practice. To avoid the influence of leg length discrepancies and pelvic inclination [19,20], the patients were required to try to straighten both lower extremities during preoperative radiography of the entire spine, and the S1 tilt was measured precisely, which should be consistent with the angle between the crossbar and the sacrum intraoperatively. On this basis, the angle between the upper endplate of the UIV and the crossbar should be consistent with the ideal postoperative UIV tilt calculated preoperatively. The related parameters were measured or calculated by the formula pre, intra-, or postoperatively.

### 2.5. Statistical Analysis

All parameters are expressed as the mean ± SD (standard deviation). Data with a normal distribution were assessed with the Shapiro–Wilk test. Correlations were analyzed using the Pearson or Spearman correlation coefficient, and simple linear regressions were simultaneously conducted. All statistical analyses were performed using SPSS software (version 25; IBM Corp., Armonk, NY, USA). A two-tailed *p* value < 0.05 was set as the level of significance.

## 3. Results

A total of 76 patients were recruited in the retrospective study and were divided into three groups based on postoperative LSB: the whole group, the LSI group, and the LSB group, with mean ages of 14.16 ± 3.15 years, 14.42 ± 4.43 years, and 14.02 ± 2.27 years, respectively. The majority of patients were female (78.95%, 88.46%, and 74.0%, respectively). The incidence of LSI in this retrospective study was 34.21%. The demographics of scoliosis patients and measured or calculated parameters are illustrated in Table 1.

Analysis of the correlation between the preoperative T1 tilt and preoperative CA and that between the postoperative T1 tilt and postoperative CA showed weak or no obvious correlations; the correlation coefficients and *p* values for each of the three groups are shown in Table 2, Table 3, and Table 4, respectively. However, the change in T1 tilt after correction surgery was significantly related to the change in the CA, with correlation coefficients in the whole, LSI, and LSB groups of 0.990, 0.980, and 0.992, respectively (*p* < 0.001).

Analysis of the correlation between the preoperative UIV tilt and preoperative CA and that between the postoperative UIV tilt and postoperative CA showed weak or no obvious correlations; the correlation coefficients and *p* values for each of the three groups are shown in Table 2, Table 3, and Table 4, respectively. The change in UIV tilt after correction surgery was significantly related to the change in the CA, with correlation coefficients in the whole, LSI, and LSB groups of 0.646, 0.466, and 0.692, respectively (*p* < 0.01). These coefficients were smaller than those found for T1.

The correlation analysis between the difference in the actual and ideal postoperative UIV tilt values and the postoperative CA showed a significant correlation between them, with correlation coefficients in the whole, LSI, and LSB groups of 0.981, 0.982, and 0.953, respectively (*p* < 0.001) (Table 2, Table 3, and Table 4).

To verify the feasibility of this method in clinical practice, 13 scoliosis patients were enrolled in a prospective study. The majority of patients were female (84.62%). Based on this correction method, good postoperative LSB (also defined as |CA| < 3 degrees) was achieved in all patients. Demographics of scoliosis patients and measured or calculated parameters are illustrated in Table 1. Consistent with the retrospective study, the correlation analysis showed a significant correlation between the difference in the actual and ideal postoperative UIV tilt values and the postoperative CA, with a correlation coefficient of 0.939 (*p* < 0.001). The actual postoperative UIV tilt was significantly correlated with the ideal postoperative UIV tilt, with a correlation coefficient of 0.966 (*p* < 0.001). The significant correlation between the intraoperative UIV tilt and the ideal postoperative UIV tilt (correlation coefficient, 0.997; *p* < 0.001), as well as between the intraoperative UIV tilt and the postoperative UIV tilt (correlation coefficient, 0.982; *p* < 0.001), suggests that the improved crossbar method can control the UIV tilt angle well during correction surgery (Table 5).

**Figure 1:**
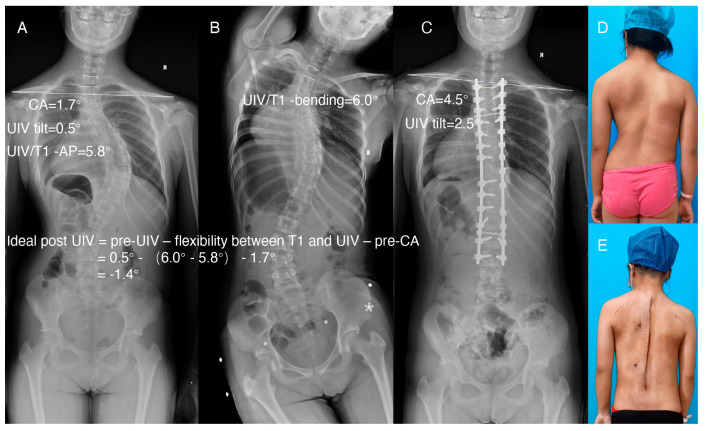
Representative LSI case in the retrospective study. (**A**) AP radiograph of the entire spine preoperatively. (**B**) Right bending. (**C**) AP radiograph of the entire spine postoperatively. (**D**,**E**) Clinical images of the same patient preoperatively and postoperatively. The ideal postoperative UIV tilt, calculated by the formula using the parameters measured in A and B, was −1.4 degrees. The actual postoperative UIV tilt was 2.5 degrees, and the postoperative CA tilt was 4.5 degrees.

**Figure 2:**
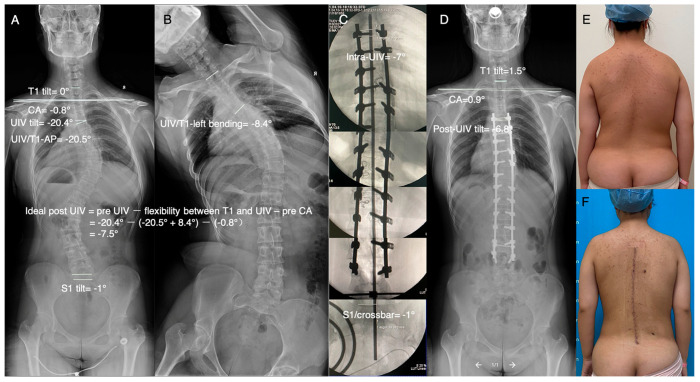
Representative case of using the improved crossbar technique to control the intraoperative UIV tilt to achieve postoperative LSB. (**A**) AP radiograph of the entire spine preoperatively. (**B**) Left bending. The ideal postoperative UIV tilt, calculated by the formula using the parameters measured in A and B, was −7.5 degrees. (**C**) Use of intraoperative fluoroscopic images and the improved crossbar technique to control the intraoperative UIV tilt, which was −7 degrees. (**D**) AP radiograph of the entire spine postoperatively. The actual postoperative UIV tilt was −6.8 degrees, and the postoperative CA tilt was 0.9 degrees. (**E**,**F**) Clinical images of the same patient preoperatively and postoperatively.

**Figure 3:**
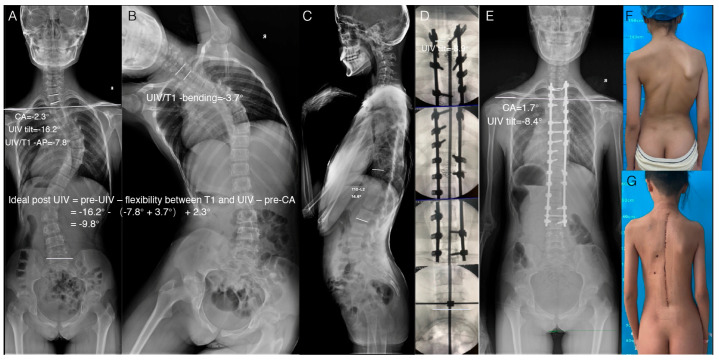
Representative case of using the improved crossbar technique to control the intraoperative UIV tilt to achieve postoperative LSB. (**A**) AP radiograph of the entire spine preoperatively. (**B**) Left bending. The ideal postoperative UIV tilt, calculated by the formula using the parameters measured in A and B, was −9.8 degrees. (**C**) Sagittal radiograph of the entire spine preoperatively. T10-L2 kyphosis was 14.6°. (**D**) Use of intraoperative fluoroscopic images and the improved crossbar technique to control the intraoperative UIV tilt, which was −8.9 degrees. (**E**) AP radiograph of the entire spine postoperatively. The actual postoperative UIV tilt was −8.4 degrees, and the postoperative CA tilt was 1.7 degrees. (**F**,**G**) Clinical images of the same patient preoperatively and postoperatively.

A representative LSI case in the retrospective study is shown in Figure 1. Two representative LSB cases in the prospective study are shown in Figure 2 and Figure 3. The ideal postoperative UIV tilt was predicted by the formula mentioned above, and the improved crossbar method was used intraoperatively to achieve the same intraoperative UIV tilt as the predicted ideal postoperative UIV tilt, resulting in satisfactory LSB in the patient.

## 4. Discussion

Many techniques for preventing LSI have been reported, such as appropriate correction rate of the MT curve [1,21,22] and selection of an appropriate UIV [9,13,14,23]; however, this complication remains prevalent in scoliosis patients, reported in at least 25% of cases [1,2], and how to achieve LSB postoperatively remains unclear [8].

Our study found that the change in T1 tilt after correction surgery was significantly related to the change in the CA, with correlation coefficients greater than 0.98, which suggests a close connection between T1 and the clavicle. Then, the ideal T1 tilt can be predicted before surgery to prevent LSI. However, in clinical work, we do not routinely fuse T1 but instead fuse T2 or below [9,12]. Therefore, the question of the factors influencing T1 tilt should be speculated. First, the existing preoperative CA and UIV tilt need to be considered [15,24]. Second, the flexibility between T1 and the UIV needs to be considered. If the selected UIV is T1, the preoperative CA can be directly subtracted from the preoperative T1 tilt, yielding the angle at which the postoperative UIV should be reserved. In the current retrospective study, the change in UIV tilt after correction surgery was significantly related to the change in the CA, while the correlation coefficients were smaller than those for T1, which may be related to the flexibility between UIV and T1 [15].

Traditionally, the ideal orthopedic effect is to correct scoliosis as much as possible and have a good overall balance. However, the goal of leveling the upper thoracic spine does not appear to guarantee clinically balanced shoulders or clavicles [8]. Therefore, how to place the UIV as flat as possible on the premise of ensuring shoulder balance requires good calculation and evaluation. We have found in practice that if the right side of the UIV is high before surgery, and if we place the UIV as flat as possible, and the left side needs to be raised, we need to take into account the maximum compensatory ability between T1 and UIV to bend to the left; if the UIV is high on the left side before surgery, in order to put the UIV as flat as possible, the right side needs to be raised, and we need to refer to the maximum compensatory ability between T1 and UIV to bend to the right. Then, the ideal UIV tilt angle can be precisely calculated before the operation by determining the preoperative UIV tilt and subtracting the reserve flexibility between the T1 and the UIV and the already existing CA, as described by the formula mentioned above.

The results of the correlation analysis between the difference in the actual and ideal postoperative UIV tilt values and the postoperative CA in the retrospective study confirmed that our speculation was correct. If the UIV is positioned to achieve the ideal postoperative UIV tilt, LSB should be achieved; otherwise, the difference between the actual postoperative UIV tilt and the ideal postoperative UIV tilt should be the value of the postoperative CA.

The current prospective study verified the feasibility of this method in clinical practice. Consistent with the retrospective study, the correlation analysis showed a significant correlation between the difference in the actual and ideal postoperative UIV tilt values and the postoperative CA, as well as between the actual postoperative UIV tilt and the ideal postoperative UIV tilt, which resulted in satisfactory postoperative LSB in all enrolled scoliosis patients. The significant correlation between the intraoperative UIV tilt and the ideal postoperative UIV tilt, as well as between the intraoperative UIV tilt and the actual postoperative UIV tilt, suggests that the improved crossbar method can control the intraoperative UIV tilt well during correction surgery.

The advantage of this method is that the ideal postoperative UIV tilt predicted by the formula is calculated when the maximum compensation is reached between the UIV and T1 vertebra, so the LSB problem is digitalized. The difference between the ideal postoperative UIV tilt and the actual postoperative UIV tilt will be directly reflected by the postoperative CA, so the operation can be accurately performed by adjusting the intraoperative UIV tilt to be consistent with the predicted ideal postoperative UIV tilt using the improved crossbar method to indirectly affect the postoperative T1 tilt, more reliably resulting in LSB after correction surgery.

Previous studies have shown that some of the patients with shoulder imbalance immediately after surgery had an improvement in shoulder balance during the 2-year follow-up period, which may be associated with the postoperative adding-on or trunk shift phenomenon [3,25]. Although patients with LSI do not require revision surgery, LSI should be avoided because it may not only affect the patients’ appearance but may also aggravate the postoperative adding-on or trunk shift phenomenon during the follow-up period [3]. Therefore, it is necessary to optimize shoulder balance during correction maneuvers intraoperatively to prevent these problems, and using our method to achieve lateral shoulder balance immediately after surgery may be an option to avoid such problems.

## Figures and Tables

**Table 1:** Demographics of scoliosis patients and measured or calculated parameters.

Variables	Retrospective Study			Prospective Study
	Whole Group	LSI	LSB	
Age (y)	14.16 ± 3.15	14.42 ± 4.43	14.02 ± 2.27	13.46 ± 2.26
Sex [*n* (%)]				
Male	16 (21.05)	3 (11.54)	13 (26.0)	2 (15.38)
Female	60 (78.95)	23 (88.46)	37 (74.0)	11 (84.62)
UIV [*n* (%)]				
T2	45 (59.21)	18 (69.23)	27 (54.0)	4 (30.77)
T3	16 (21.05)	4 (15.38)	12 (24.0)	3 (23.08)
T4	9 (11.84)	2 (7.70)	7 (14.0)	4 (30.77)
T5	6 (7.90)	2 (7.69)	4 (8.0)	2 (15.38)
Pre-CA	−1.30 ± 3.30	−0.56 ± 2.15	−1.69 ± 3.72	−1.02 ± 1.95
Pre-UIV	−3.35 ± 13.44	−4.31 ± 15.22	−2.85 ± 12.55	−8.73 ± 10.70
(T1/UIV Cobb) in AP	−3.40 ± 10.36	−5.12 ± 10.71	−2.50 ± 10.17	−8.59 ± 8.53
(T1/UIV Cobb) in bending	−1.85 ± 5.62	−2.68 ± 6.32	−1.42 ± 5.23	−2.96 ± 4.95
Post-CA	1.51 ± 2.38	3.45 ± 2.61	0.51 ± 1.47	0.68 ± 1.27
Post-UIV	0.89 ± 8.09	1.76 ± 10.20	0.43 ± 6.81	−1.48 ± 5.35
Ideal UIV	−0.50 ± 8.38	−1.31 ± 11.09	−0.08 ± 6.64	−2.08 ± 5.34
(Post-Ideal) UIV	1.47 ± 2.44	3.53 ± 2.58	0.40 ± 1.51	0.60 ± 1.39
(Post-Pre) CA	2.82 ± 3.64	4.01 ± 2.44	2.20 ± 4.02	1.70 ± 1.68
Pre-T1	0.37 ± 6.12	−0.78 ± 7.25	0.96 ± 5.42	0.38 ± 5.68
Post-T1	3.13 ± 5.66	3.30 ± 7.81	3.05 ± 4.23	1.97 ± 4.98
(Post-Pre) T1	2.77 ± 3.68	4.08 ± 2.58	2.09 ± 3.99	1.58 ± 1.91
(Post-Pre) UIV	4.24 ± 8.03	6.07 ± 7.47	3.29 ± 8.21	7.25 ± 5.91
Intra-UIV	-	-	-	−1.81 ± 5.21

CA, clavicle angle; UIV, upper instrumented vertebra; LSI, lateral shoulder imbalance; LSB, lateral shoulder balance; Pre, preoperative; AP, anteroposterior; Post, postoperative; Intra, intraoperative.

**Table 2:** Retrospective correlation analysis of the measured or calculated parameters in the whole group.

Variables	Correlation Coefficient	*p* Value
Pre-T1 vs. Pre-CA	0.428 **	0.000
Post-T1 vs. Post-CA	0.286 *	0.012
(Post-Pre) T1 vs. (Post-Pre) CA	0.990 **	0.000
Pre-UIV vs. Pre-CA	0.411 **	0.000
Post-UIV vs. Post-CA	0.238 *	0.039
(Post-Pre) UIV vs. (Post-Pre) CA	0.646 **	0.000
Pre-T1 vs. Pre-UIV	0.339 **	0.003
Post-T1 vs. Post-UIV	0.368 **	0.001
(Post-Pre) UIV vs. (Post-Pre) T1	0.617 **	0.000
(Post-Ideal) UIV vs. Post-CA	0.981 **	0.000

CA, clavicle angle; UIV, upper instrumented vertebra; Pre, preoperative; Post, postoperative. * *p* < 0.05, ** *p* < 0.01.

**Table 3:** Retrospective correlation analysis of the measured or calculated parameters in the LSI group.

Variables	Correlation Coefficient	*p* Value
Pre-T1 vs. Pre-CA	0.019	0.928
Post-T1 vs. Post-CA	0.296	0.142
(Post-Pre) T1 vs. (Post-Pre) CA	0.980 **	0.000
Pre-UIV vs. Pre-CA	0.392 *	0.048
Post-UIV vs. Post-CA	0.211	0.301
(Post-Pre) UIV vs. (Post-Pre) CA	0.466 *	0.016
Pre-T1 vs. Pre-UIV	0.025	0.904
Post-T1 vs. Post-UIV	0.182	0.373
(Post-Pre) UIV vs. (Post-Pre) T1	0.430 *	0.028
(Post-Ideal) UIV vs. Post-CA	0.982 **	0.000

LSI, lateral shoulder imbalance; CA, clavicle angle; UIV, upper instrumented vertebra; Pre, preoperative; Post, postoperative. * *p* < 0.05, ** *p* < 0.01.

**Table 4:** Retrospective correlation analysis of the measured or calculated parameters in the LSB group.

Variables	Correlation Coefficient	*p* Value
Pre-T1 vs. Pre-CA	0.682 **	0.000
Post-T1 vs. Post-CA	0.413 **	0.003
(Post-Pre) T1 vs. (Post-Pre) CA	0.992 **	0.000
Pre-UIV vs. Pre-CA	0.469 **	0.001
Post-UIV vs. Post-CA	0.276	0.052
(Post-Pre) UIV vs. (Post-Pre) CA	0.692 **	0.000
Pre-T1 vs. Pre-UIV	0.635 **	0.000
Post-T1 vs. Post-UIV	0.635 **	0.000
(Post-Pre) UIV vs. (Post-Pre) T1	0.663 **	0.000
(Post-Ideal) UIV vs. Post-CA	0.953 **	0.000

LSB, lateral shoulder balance; CA, clavicle angle; UIV, upper instrumented vertebra; Pre, preoperative; Post, postoperative. ** *p* < 0.01.

**Table 5:** Prospective correlation analysis of the measured or calculated parameters in the clinical application.

Variables	Correlation Coefficient	*p* Value
Pre-T1 vs. Pre-CA	0.702 **	0.007
Post-T1 vs. Post-CA	0.425	0.148
(Post-Pre) T1 vs. (Post-Pre) CA	0.976 **	0.000
Pre-UIV vs. Pre-CA	0.666 *	0.013
Post-UIV vs. Post-CA	0.198	0.517
(Post-Pre) UIV vs. (Post-Pre) CA	0.577 *	0.039
Pre-T1 vs. Pre-UIV	0.746 **	0.003
Post-T1 vs. Post-UIV	0.697 **	0.008
(Post-Pre) UIV vs. (Post-Pre) T1	0.587 *	0.035
(Post-Ideal) UIV vs. Post-CA	0.939 **	0.000
Ideal UIV vs. Post-UIV	0.966 **	0.000
Ideal UIV vs. Intra-UIV	0.997 **	0.000
Post-UIV vs. Intra-UIV	0.982 **	0.000

CA, clavicle angle; UIV, upper instrumented vertebra; Pre, preoperative; Post, postoperative; Intra, intraoperative. * *p* < 0.05, ** *p* < 0.01.

## Data Availability

Not applicable.
